# *Tomato Brown Rugose Fruit Virus*: Seed Transmission Rate and Efficacy of Different Seed Disinfection Treatments †

**DOI:** 10.3390/plants9111615

**Published:** 2020-11-20

**Authors:** Salvatore Davino, Andrea Giovanni Caruso, Sofia Bertacca, Stefano Barone, Stefano Panno

**Affiliations:** 1 Department of Agricultural, Food and Forest Sciences (SAAF), University of Palermo, Viale delle Scienze, 90128 Palermo, Italy; andreagiovanni.caruso@unipa.it (A.G.C.); sofia.bertacca@community.unipa.it (S.B.); stefano.barone@unipa.it (S.B.); 2 Institute for Sustainable Plant Protection, National Research Council (IPSP-CNR), Strada delle Cacce, 73-10135 Turin, Italy; 3 Department of Biological, Chemical and Pharmaceutical Sciences and Technologies (STEBICEF), University of Palermo, Via Archirafi, 18-90123 Palermo, Italy

**Keywords:** ToBRFV, seed transmission, seed disinfection

## Abstract

Tomato brown rugose fruit virus (ToBRFV) is a highly infectious virus, that is becoming a threat to tomato production worldwide. In this work we evaluated the localization of ToBRFV particles in tomato seeds, its seed transmission rate and efficacy of disinfection, and the effects of different thermal- and chemical-based treatments on ToBRFV-infected seeds’ germination. Analyses demonstrated that ToBRFV was located in the seed coat, sometime in the endosperm, but never in the embryo; its transmission from infected seeds to plantlets occurs by micro-lesions during the germination. The ToBRFV seed transmission rate was 2.8% in cotyledons and 1.8% in the third true leaf. Regarding the different disinfection treatments, they returned 100% of germination at 14 days post-treatment (dpt), except for the treatment with 2% hydrochloric acid +1.5% sodium hypochlorite for 24 h, for which no seed germinated after 14 dpt. All treatments have the ability to inactivate ToBRFV, but in six out of seven treatments ToBRFV was still detectable by RT-qPCR. These results raise many questions about the correct way to carry out diagnosis at customs. To our knowledge, this is the first study on the effective localization of ToBRFV particles in seeds.

## 1. Introduction

Tomato (*Solanum lycopersicum* L., family Solanaceae) has become, in the past fifty years, one of the most important and extensively grown horticultural crops worldwide. In 2018, more than 182 million tons of tomato were produced globally [1]. China is the most important tomato producer (over 61 million tons), followed by India, the United States of America and Turkey, while Italy and Spain are the major tomato producers in Europe (over 5.7 million and 4.7 million tons, respectively) [1]. In the decade between 2008 and 2018, the global tomato production increased by more than 40 million tons [1]. Tomatoes and many other vegetable crops are continually exposed to new biotic factors, such as viral diseases which cause new phytosanitary emergencies. Phytoviruses are difficult to manage, due to their short replication time, frequent mutation/recombination events, host plant preference and, more importantly, different transmission methods [2,3,4].

More than 200 plant viruses are transmitted by seed. The frequency of seed transmission is higher in the *Potyvirus*, *Potexvirus*, *Nepovirus*, *Ilarvirus*, *Tobamovirus*, *Potexvirus*, *Cucumovirus* and *Bromovirus* genera, which infect important vegetable crops such as tomato. Some viruses are highly specific and infect only one or at least two plant species, or only the species within one family. Other viruses, such as tobacco mosaic virus (TMV) or cucumber mosaic virus (CMV), can infect a wide range of plant species belonging to different families, including herbaceous and woody plants [5]. Despite the high number of viruses that are transmitted by seed, only stable viruses, such as TMV, are localized on the seed coat, and seedling infection occurs mainly by mechanical transmission, for example during transplanting [6,7].

The majority of viral particles are inactivated in the seed coat and embryo during seed maturation, and only a small number of viral particles are able to infect the seed. Virus inactivation during seed maturation has been demonstrated in several cases, such as for alfalfa mosaic virus (AMV) [8]. Furthermore, seed-borne transmitted viruses never come into contact with the emerging seedling if the seed coat separates from the seedling during germination [9]. The transmission of viruses that are localized on the external seed coat is therefore a rare phenomenon, probably because only a few plant viruses are sufficiently stable to withstand the environmental exposure (i.e., dehydration, harvest and storage), and viable viruses are not able to infect the seedling until transplantation or mechanical inoculation caused by handling [5,10].

All these key factors play a crucial role in understanding the virus. This is the case for the recent tomato brown rugose fruit virus (ToBRFV) outbreaks in different countries.

Tomato brown rugose fruit virus belongs to the genus *Tobamovirus* (family *Virgaviridae*), which represents one of the biggest genera of its family, due to the high number of viral species. Differently from other members of this family, tobamoviruses have an undivided genome [11]. ToBRFV has the typical genome organization of the *Tobamovirus* genus. A single-stranded positive-sense RNA (+ssRNA) molecule of approximately 6400 nucleotides (nt) contains four open reading frames (ORFs), encoding the following: two replication-related protein complexes of 126 and 183 kDa (ORF1a and ORF1b, respectively), where the second protein is expressed by the partial suppression of the stop codon; the movement protein (MP) of ca. 30 kDa (ORF2); the coat protein of ca. 17.5 kDa (ORF3), expressed via the 3′-coterminal sub-genomic RNAs [12].

The symptoms caused by ToBRFV infection consist of tomato leaves’ interveinal yellowing, deformation and mosaic staining, young leaves’ deformation and necrosis, sepal necrosis and deformation, and young fruits’ discolouration, deformation, marbling and necrosis.

ToBRFV transmission is mainly mechanical, but it can also occur via contaminated seeds or fruits over long distances, such as for other common tobamoviruses. The mechanical transmission of this new pathogen within crops can occur through direct contact with infected plants [12], or infected sap from different surfaces (operator, clothing, pots, packaging, consumption of tomatoes coming from a different crop, transport equipment, working tools, nutrient solutions) [13], propagation materials (grafts, cuttings), bumblebees and seeds [14]. After harvesting, ToBRFV inoculum can also be harbored in several surfaces and materials of a greenhouse, such as wires, glass, concrete and soil [15].

All ToBRFV isolates that have been reported in different affected areas are genetically closely related, suggesting that they originate from a unique common ToBRFV ancestor [15]. This scenario reinforces the hypothesis that the dissemination of this virus was caused by seed transmission from one country to another. Tobamoviruses are transmitted with low frequency by mechanical inoculation through the contact of emerging cotyledons and infected seed coats. However, the presence of a few infected plants can have a huge impact on high intensity glasshouse production [5]. Until now, there have been no studies that confirm ToBRFV seed-transmission, because this virus appears to be confined only in the seed coat and not in the endosperm or embryo [15]. Seed transmission probably occurs as a result of the contact between the germinating seedling and the virus-contaminated seed coat, followed by localized mechanical spread.

ToBRFV was identified and described for the first time in 2016 by Salem et al. [12] in tomato plants grown in a greenhouse in Jordan, and in 2017 on tomato plants harboring the *Tm-2*^2^ gene in Israel [16]. To date, ToBRFV has been detected in tomato plants in Mexico [17], the United States of America (California) [18], Germany [19], Italy [3], Palestine [20], Turkey [21], United Kingdom [22], Greece [23], China [24], Spain [25], Holland [26], France [27], Czech Republic [28] and Cyprus [29].

The virus has also been reported in sweet pepper plants grown under plastic houses in Jordan and in greenhouses in Italy [30,31]. However, due to ToBRFV’s ability to move though seeds and contaminated fruits [15,16], the reports available before today have probably been underestimated.

ToBRFV-tolerant or -resistant varieties are currently not known. For this reason, an integrated management system is necessary to minimize as much as possible ToBRFV dispersion, using all available technologies and virus knowledge.

Control strategies and effective viral impact mitigation programs are essential to improve the understanding of ToBRFV dispersion. The aim of the current study was to evaluate ToBRFV viral particles’ localization on tomato seeds, the seed-transmission rate, the efficacy of different disinfection treatments (chemical and physical) on ToBRFV-infected seeds, and the effect of these treatments on seed germination. This information may be essential for ToBRFV management, avoiding the introduction of this virus into new countries through ToBRFV-infected seeds.

## 2. Materials and Methods

### 2.1. Preparation of Infected ToBRFV-Seeds

Sap extract of ToBRFV ToB-SIC01/19 isolate, Acc. No. MN167466 [4], was mechanically inoculated into 10 tomato-cherry plants (*Solanum lycopersicum* L.) with the *Tm-2^2^* resistance gene, kindly provided by an anonymous seed company.

About 200 mg of fresh leaf tissue from an infected plant were ground in a mortar with 6 mL of phosphate buffer pH 7 (0.2 M NaH_2_PO_4_, 0.2 M Na_2_HPO_4_ x 7H_2_O), distributed and rubbed on plant leaf surfaces previously sprinkled with *Carborundum* (320 mesh), which causes micro-lesions and facilitates the virions’ entry. The inoculated plants were grown in sterilized soil with a photoperiod of 14 h of light and an air temperature of 28/20 °C day/night in an insect-proof glasshouse [32]. Severe phytosanitary measures, isolation, insect control, and careful handling were applied to avoid the accidental virus spread. The symptoms of the viral infection were recorded every 15 days, and the presence of ToBRFV was evaluated in all plants by one-step RT-qPCR [4] at 30 dpi. Total RNA was extracted from all the collected samples using the Plant RNA/DNA Purification Kit (Norgen Biotek Corp., Thorold, ON, Canada) and following the manufacturer’s instruction. Total RNA extracts were re-suspended in 30 μL of RNase-free water and the concentration was adjusted to approximately 10 ng/μL. The RT-qPCR assay was performed with a Rotor-Gene Q2plex HRM Platform Thermal Cycler (Qiagen, Hilden, Germany) in a reaction mix of 12 μL final volume, containing 1 μL of total RNA extract (∼10 ng/μL), 0.5 μM of the forward primer ToB5520F (5′-GTAAGGCTTGCAAAATTTCGTTCG-3′), 0.5 μM of the reverse primer ToB5598R (5′-CTTTGGTTTTTGTCTGGTTTCGG-3′), 0.25 mM of specific probe (5′-FAM GTTTAGTAGTAAAAGTGAGAAT-MGB-3′), 0.5 µl of RNase Inhibitor (Applied Biosystems, Foster City, CA, USA), 6 μL of 2X QuantiNova Probe RT-PCR Master Mix, 0.2 μL of QN Probe RT-Mix and H_2_O DEPC to reach final volume [4].

The plants were grown until the tomato fruit’s complete maturation of the first 3 stages. In total, 50 tomato fruits per plant were collected at the end of the third month. Tomato fruits were cut in two parts and seeds were collected. The seeds obtained from different fruits were mixed in order to constitute a unique lot. The retrieved seeds were transferred to a glass baker with 500 mL of sterile H_2_O at a temperature of 25 °C to dissolve excess pulp. After 24 h, the seeds were recovered, washed in a sieve for 10 min and dried at room temperature in tissue paper for one week. In total, 250 seeds were analyzed by one step RT-qPCR, as previously described, to ascertain the effective presence of ToBRFV.

### 2.2. ToBRFV Localization on Tomato Seed

A total of 2000 tomato seeds was divided into two subsamples: a batch of 1000 seeds, named Bt (treated batch), was cleaned by incubation in 10% (*w/v*) trisodium phosphate for 180 min, washed with sterile water for 30 min, and dried at room temperature for 48 h. A second batch of 1000 seeds, named Bnt (not treated batch), was used as control.

Five hundred seeds of Bt and five hundred seeds of Bnt were pre-germinated in Petri dishes on moist tissue paper at 25 °C for 7 days to physically separate the seed coat from the embryo. The seed coats of each germinating seed were separated from the root+cotyledons with a pair of fine-point forceps, cleaned with a 10% (*w/v*) trisodium phosphate solution, and collected by type in groups of ten in 1.5 mL tubes. The separated subsamples were cleaned in 10% (*w/v*) trisodium phosphate for 1 min and washed with sterile distilled water to remove any potential surface virus particle. The following subsamples were obtained: 50 Bt seed coats (Bt-T), 50 Bt root+cotyledons (Bt-RC), 50 Bnt seed coats (Bnt-T), and 50 Bnt root+cotyledons (Bnt-RC), for a total of 200 subsamples. Total RNA was extracted from the 200 subsamples and used for one step RT-qPCR, as described by Panno et al. [4].

ToBRFV presence or absence in the seeds of Bt and Bnt batches was assessed according to the Ct values obtained by one step RT-qPCR. The obtained results suggested we apply the Fisher exact test [33], in which the inefficacy of the treatment is the null hypothesis to test. The test was applied both for the seed coats and the root+cotyledons.

### 2.3. ToBRFV Seed Transmission Rate

ToBRFV’s seed transmission rate to tomato seedlings was evaluated using approximately 500 Bnt seeds that were sown in sterile 40-plug trays containing sterilized soil substrate with a single seed per plug. The plug trays were placed in a phytotron with a photoperiod of 14 h light and a target air temperature of 28/20 °C day/night. One cotyledon of each plant was collected. The obtained 500 samples were analyzed by one step RT-qPCR [4] to ascertain the effective presence of ToBRFV. A cotyledon slice of 0.4 mm of each sample was directly placed in a 1.5 mL tube containing 0.5 mL of Glycine buffer (EDTA 1 mM, NaCl 0.05 M, Glycine 0.1 M), vortexed for 30 s and heated at 95 °C for 10 min; three microlitres were used for molecular analysis. Sampling was repeated two weeks later, collecting the third true leaf. Furthermore, in this case, samples were analyzed as previously described.

### 2.4. Effect of Seed Treatments on Tomato Seed Germination and Efficacy of Different Disinfection Methods

To determine the efficacy of seed disinfection treatments against ToBRFV, four thermal-based treatments and four chemical-based treatments were tested. For each experiment, a seed batch of 100 seeds was used.

#### 2.4.1. Thermal-Based Treatments on ToBRFV-Infected Seeds

Four different thermal-based treatments were applied on ToBRFV-infected seeds, according to the following protocols: (i) seeds heated for 24 h at 80 °C (ST-80); (ii) seeds heated for 48 h at 75 °C (ST-75); (iii) seeds heated for 96 h at 70 °C (ST-70); (iv) seeds heated for 120 h at 65 °C (ST-65). The seeds were pre-germinated in Petri dishes on moist tissue paper at 25 °C after the different treatments to understand if each treatment affects seed germination, in terms of percentage and time. Each sample was collected in a 1.5 mL Eppendorf tube, and homogenized with extraction buffer (1.3 g sodium sulphite anhydrous, 20 g polyvinylpyrrolidone MW 24-40,000, 2 g powdered egg (chicken) albumin Grade II, 20 g Tween-20 in one L of distilled water, pH 7.4). Quantities of 5 μL of each sample were spotted on a 1 cm^2^ Hybond^®^-N+ hybridization membrane (GE Healthcare, Chicago, IL, USA), dried at room temperature for 5 min and placed in a 1.5 mL tube containing 0.5 mL of glycine buffer (1 mM EDTA, 0.05 M NaCl, 0.1 M Glycine). The tubes were vortexed for 30 s and heated at 95 °C for 10 min. Measures of 3 μL were used for one step RT-qPCR [4]. The homogenized seedlings that gave a positive signal in the RT-qPCR were collected in groups of 10, and mechanically inoculated in tomato plants cv. Marmande, to understand if the virus was not only detectable but also infectious. The homogenized samples of each group of 10 seedlings were distributed and rubbed on the plant leaf surfaces, previously sprinkled with Carborundum (320 mesh). The inoculated plants were grown in controlled conditions in an insect-proof glasshouse. The symptoms of viral infection were recorded every 7 days, and the presence of ToBRFV was evaluated in all plants by RT-qPCR at 30 dpi, after total RNA extraction using the Plant RNA/DNA Purification Kit (Norgen Biotek Corp., Thorold, ON, Canada) following the manufacturer’s instruction. A measure of 1 μL of total RNA was used for RT-qPCR [4].

#### 2.4.2. Chemical-Based Treatments on ToBRFV-Infected Seeds

Four different chemical-based treatments were tested to evaluate the possibility of eradicating ToBRFV from seeds, according to the following protocols: (i) seeds submerged in 10% trisodium phosphate solution for 180 min (ST-P); (ii) seeds submerged in 4% hydrogen peroxide for 30 min (ST-H); (iii) seeds submerged in 2% hydrochloric acid +1.5% sodium hypochlorite for 24 h (ST-A); (iv) seeds submerged in 2.5% sodium hypochlorite solution for 15 min (ST-S). At the end of each treatment the seeds were washed three times for 5 min with sterile distilled water and dried on sterile absorbent paper for 1 week. Furthermore, in this case, the seeds were pre-germinated after the different treatments. Each obtained seedling was used for molecular analysis following the protocol described above. The bioassay was conducted as described in Section 2.4.1. All samples that gave a positive signal in the RT-qPCR were mechanically inoculated in tomato plants cv. Marmande, in order to understand if the virus was detectable but not infectious. The Ct values obtained by RT-qPCR evaluated on the 8 samples out of 100 permitted us to establish whether the seedling was infected or not. Point estimates of the proportions of infected seedlings could be easily devised. In addition, the confidence intervals of the proportions could be calculated by classic methods [34].

## 3. Results

### 3.1. Preparation of Infected ToBRFV-Seeds

Typical ToBRFV symptoms on leaves, consisting of mosaic, interveinal yellowing and deformations, appeared at 15 dpi. RT-qPCR [4] performed at 30 dpi gave positive results in all the inoculated tomato plants, with Ct values ranging from 14.32 to 16.84 (data not showed). In total, 50 tomato fruits per plants were collected, and the seeds were retrieved. A subsample, constituted by 250 seeds and analyzed by RT-qPCR as previously described, gave positive results with a Ct value of 14.03, confirming the effective presence of ToBRFV in the seeds derived from the infected plants.

### 3.2. ToBRFV Localization on Tomato Seed

Totals of 500 treated seeds (Bt) and 500 not treated seeds (Bnt) were pre-germinated in Petri dishes. All seeds produced seedlings after 7 days at 25 °C. Five hundred seedlings originated from Bt and five hundred from Bnt were separated into seed coats and root+cotyledons, and were collected by type in groups of ten, for a total of 100 samples (50 Bt + 50 Bnt), respectively. The analysis performed on the Bnt seed coats (Bnt-T) showed 100% ToBRFV infection, and all samples gave positive results with Ct values ranging from 30.07 to 33.56. Regarding Bnt root+cotyledons (Bnt-RC) samples, only two samples (4%) gave a positive signal, with Ct values of 33.49 and 33.79. In the case of Bt seed coats (Bt-T) samples, only one sample (2%) gave a positive result, with a Ct value of 32.66. Bt root+cotyledons (Bt-RC) samples were all negative to RT-qPCR (Table 1).

The results obtained by the Fisher exact test applied to Bt/Bnt seed coats and root+cotyledons are reported in a contingency table (Table 2).

Applying the Fisher exact test, the probability of observing the scenario in the null hypothesis of inefficacy of the treatment is *p* = 5.05 × 10^−28^, which is extremely low. However, the most extreme scenario is shown in Table 3, with a probability of occurrence equal to *p* = 9.91 × 10^−30^.

The sum of the two probabilities gave a *p*-value for the hypothesis test equal to 5.15 × 10^−28^, which allows us to reject the null hypothesis of inefficacy of the treatment. The same hypothesis test was made for root+cotyledons and the results are reported in the following contingency table (Table 4).

This scenario has a probability of occurrence in the null hypothesis of inefficacy of the treatment equal to *p* = 0.25. There are no scenarios more extreme than this. Therefore, the efficacy of the treatment is not very significant in root+cotyledons, but is rather likely.

### 3.3. ToBRFV Seed Transmission Rate

In order to verify the ToBRFV seed transmission rate to tomato seedlings, the cotyledons and the third true leaf taken from the Bnt seeds were analyzed. Of 500 cotyledons samples, 14 (2.8%) gave positive results in the RT-qPCR, with Ct values ranging from 27.57 to 34.45, and only 9 from 500 third true leaf samples (1.8%) gave positive signals via RT-qPCR, with Ct values ranging from 32.56 to 34.90 (Table 5).

### 3.4. Effect of Seeds Treatment on Tomato Seed Germination and Efficacy of Different Disinfection Methods

The percentage of seed germination related to each treatment is reported in Table 6. All the seeds germinated within two weeks, except for the seeds of the ST-A treatment, which did not germinate. Therefore, seven out of eight treatments proved to be applicable without compromising germination.

Thermal-based procedure: with the ST-80 treatment (i), 100% of seed germination was obtained after 7 days post-treatment, and 60% of the samples analyzed by RT-qPCR gave positive results, with Ct values ranging from 25.83 to 27.68; after 7 days with the ST-75 treatment (ii), only the 67% of the seeds germinated, while after 14 days all seeds germinated; 80 out of 100 samples gave positive results under RT-qPCR, with Ct values ranging from 20.07 to 26.04; with the ST-70 treatment (iii), seed germination percentage reached 89% after 7 days, and 100% after 2 weeks; all samples gave positive results via RT-qPCR, with Ct values ranging from 24.54 to 26.45; finally, with the ST-65 treatment (iv), the germination percentage reached 100% after 7 days post-treatment, and all samples gave positive results via RT-qPCR, with Ct values ranging from 23.43 to 28.38 (Supplementary Table S1).

As reported in the Materials and Methods, the samples that gave positive results under RT-qPCR were grouped in lots of 10, and were mechanically inoculated in a total of 34 tomato plants. Positive samples after the ST-80 and ST-75 treatments were inoculated in six and eight tomato plants, respectively. At 30 dpi, these plants were individually analyzed and gave negative results under RT-qPCR. The positive samples after the ST-70 and ST-65 treatments were inoculated in 10 plants each. At 30 dpi, for the ST-70 treatment, all plants gave negative results under RT-qPCR, while for the ST-65 treatment, 2 out of 10 plants (20%) were positive.

Chemical-based procedure: with the ST-P treatment (i), the seed germination percentage reached 94% after 7 days and 100% after 2 weeks; 3 of 100 samples gave positive results via RT-qPCR, with Ct values of 25.83, 26.80 and 27.68; the ST-H treatment (ii) showed 100% seed germination after 7 days and all samples gave positive results under RT-qPCR, with Ct values ranging from 24.94 to 27.31; after the ST-A treatment (iii), no seed germinated after 14 days post-seeding and, for this reason, the molecular analyses were not performed; finally, with the ST-S treatment (iv), seed germination reached 88% after one week and 100% after two weeks post-treatment, and all samples gave negative results via RT-qPCR (Supplementary Table S1).

The homogenized seedlings that gave positive results under RT-qPCR were grouped in lots of 10, and mechanically inoculated in tomato plants, except for the homogenized seedlings treated with the ST-P treatment, which were inoculated individually. Three and six tomato plants were inoculated with seedlings after the ST-P and ST-H treatments, respectively. At 30 dpi, no plant showed the typical ToBRFV symptoms, and the RT-qPCR analyses gave negative results for all the inoculated plants. For this reason, the bioassay was not performed.

Regarding the Ct values obtained by RT-qPCR, Table 7 shows 8 out of 100 samples as the proportion of infected seeds, with respective lower and upper bounds of the confidence intervals (confidence level 0.95) [34].

## 4. Discussion

The study of the epidemiology and of the distinct mechanisms involved in the dispersion of different pathogens represents one of the most important measures for containing a possible epidemic [32,35] and developing efficient disease management strategies [35,36]. From this perspective, the plant pathogens being spread by seed are extremely important, because this allows the pathogens to move from one country or continent to another in an extremely short time [37].

To clarify the significance of ToBRFV transmission, it is necessary to understand the difference between seed-borne and seed-transmission. Through seed-borne transmission, the viral particles are carried by the seeds (as a contaminant or in the seed coat), but normally they do not infect the germinated seedlings. On the contrary, in seed-transmission the virus is generally located in the embryo, and is able to infect the naturally germinating seedlings [10,38,39]. Generally, when the virus is carried as a contaminant on the seed surface, it can infect seedlings during the germination and first stages of growth [40].

Tobamoviruses mostly infect the seed coat and the endosperm, such as with cucumber green mottle mosaic virus (CGMMV), in which the perisperm–endosperm envelope (PEE) can be contaminated [41]. This mechanism allows the seeds to remain infectious for a long period [42]. When the seeds are contaminated, the virions that are found on the outer coats of the seed can infect the cotyledons through tiny wounds that form during the growth, although this does not occur for all tobamovirus-contaminated seeds [43]. This mechanism enables tobamoviruses to infect the seedlings which are prepared for distribution to farmers in the nurseries.

ToBRFV diffusion relies on long distance dispersion through the movement of infected seeds from one country to another, and on short distance dispersion by operators, pollinating insects, and—the most important—plant-to-plant contact that represents the most dangerous aspect for ToBRFV’s rapid spread [32].

The results of this work suggest that the virus is localized in the external teguments of the seed, although in some cases, probably depending on the viral accumulation that plays an important role in the transmission [44], it seems to be found in the endosperm, but never in the embryo. In addition, the transmission to the seedling is likely to occur through the micro-lesions that are caused during the germination and initial growth stages.

In this work we demonstrated that the transmission rate in tomato-cherry is around 2.8% to the cotyledons and 1.8% to the third true leaf. Considering ToBRFV’s high plant-to-plant transmission rate, very few infected plants are sufficient in a greenhouse to have a 100% infection rate a few months after transplant, which is enhanced by the presence of bumblebees [14] and different agronomic practices during the production cycle [32].

This study reports that ToBRFV is a seed-borne, but not seed-transmitted, virus in tomatoes. The mechanical transmission of ToBRFV from infected seeds to seedlings is very likely responsible for initiating a new infection. To our knowledge, this is the first report that demonstrates the localization of ToBRFV in the tomato seed coat, but not in the embryo.

The evidence that ToBRFV is never localized in the embryo gives the possibility of using different treatments for seed sterilization, without compromising the germination. For this reason, we tested different treatments, in order to understand their effectiveness related to the influence on germination.

All the proposed disinfection protocols allowed 100% germination 14 days after treatment, except for the treatment with 2% hydrochloric acid +1.5% sodium hypochlorite for 24 h, with which no seed germinated after 14 days. In the case of thermal-based treatments, regarding the ST-70 and ST-65 protocols, 100% of the tested seeds gave positive results via RT-qPCR assay [4], while for the ST-80 and ST-75 protocols, the virus was detected in 60% and 80% of the analyzed samples, respectively. Bioassays showed that the virus is detectable but not infectious after heat treatment, except for the ST-65 treatment, after which 20% of the samples gave a positive result to molecular analysis.

As far as chemical-based treatments are concerned, in this case all protocols also gave 100% germination after 14 days, except for the ST-A, which gave 0% germination, and for this reason it was excluded from subsequent analyses. With regard to the disinfection effectiveness, the chemical-based treatments also gave encouraging results. The ST-P protocol gave only 3% positive samples, and the ST-S protocol gave 0% virus detection, while the ST-H protocol gave 100% virus detection. Subsequent bioassays, as reported for thermal-based treatments, allowed us to ascertain that the virus was detectable but not infectious.

Analyzing the obtained results, we can conclude that all the thermal-based treatments can be used to disinfect ToBRFV-infected seeds, except for the ST-65 treatment (seeds heated for 120 h at 65 °C) which gave a positive result to the bioassay in 20% of cases, indicating that this treatment is not advisable. Regarding chemical-based treatments, all the tested treatments allowed a reliable seed disinfection, although the ST-S treatment (seeds submerged in 2.5% sodium hypochlorite solution for 15 min) would be preferable, as it guarantees 100% germination and complete seed disinfection. All seeds treated were actually negative by RT-qPCR. In this case, immediately after treatment, the virus is not detectable and not infectious.

According to these data, it is possible to imagine a scenario where the seeds’ movement between different countries represents a serious problem, since disputes may arise between seed companies and importing countries due to possible seed blockages at customs, and to the possibility of detecting the virus also in disinfected seeds. When a seed gives positive results to molecular analysis at customs, is the ToBRFV infectious or not? Is it possible to disembark the seed?

In our opinion, when the seeds arrive at customs, they should be analyzed applying the following protocol: (i) Immediate seeds analysis using RT-qPCR or loop-mediated isothermal amplification technique (LAMP) [45]. LAMP is a nucleic acid amplification method, that permits one to amplify a specific DNA/RNA region under isothermal conditions, using four to six primers that recognize between six and eight independent regions, enabling a fast, sensitive, and specific pathogen detection [46]. We do not recommend the use of the ISHI-Veg ToBRFV RT-qPCR protocol, which is performed by the International Seed Federation (ISF), as there is no scientific validation work that supports this method and no standard curve calculation has been reported, impeding the correct setting of the thermal cyclers and therefore an univocal response. This means that, especially for borderline analysis (low viral titer), it is possible to have different responses from distinct laboratories. (ii) The samples that gave negative results to molecular analysis can be disembarked, while positive samples, of which the infectious capacity cannot be ascertained by molecular analysis, must pass through bioassays. (iii) Bioassays must be performed on tomato plants that have the *Tm-2^2^* resistance gene, because only this test is able to ascertain if ToBRFV is infectious or not. Bioassays can also distinguish between ToBRFV, tobacco mosaic virus (TMV) or tomato mosaic virus (ToMV), since all RT-qPCR protocols could give a slight non-specific signal in the case of TMV and ToMV infection. Following these protocols, seeds would be blocked for at least 15 days, but good procedures are necessary. Alternatively, *Nicotiana benthamiana* plants could be inoculated, and if local lesions appear after 3–4 days, RNA can be extracted from the lesions and submitted to RT-PCR end point [20] to sequence the obtained RT-PCR product.

In conclusion, it would be useful to standardize an effective sterilization protocol to be used worldwide for seed movement from one country to another, and develop new molecular techniques, such as molecular hybridization [47,48], to be added to the existing ones and used for large-scale investigations.

## Figures and Tables

**Table 1:** Seed coats and root+cotyledons Ct values obtained by RT-qPCR of treated and not treated pre-germinated tomato seeds.

Sample ID	Ct Values				Sample ID	Ct Values			
	Bt-T	Bt-RC	Bnt-T	Bnt-RC		Bt-T	Bt-RC	Bnt-T	Bnt-RC
1	-	-	30.98	-	26	32.66	-	33.12	-
2	-	-	30.42	-	27	-	-	33.09	-
3	-	-	33.56	-	28	-	-	31.07	33.49
4	-	-	31.02	-	29	-	-	33.56	-
5	-	-	30.66	-	30	-	-	32.54	-
6	-	-	32.80	-	31	-	-	31.93	-
7	-	-	31.07	-	32	-	-	33.54	-
8	-	-	30.99	-	33	-	-	33.40	-
9	-	-	32.56	-	34	-	-	30.67	-
10	-	-	30.61	33.79	35	-	-	33.42	-
11	-	-	33.47	-	36	-	-	31.48	-
12	-	-	33.51	-	37	-	-	32.80	-
13	-	-	32.23	-	38	-	-	31.59	-
14	-	-	31.72	-	39	-	-	33.10	-
15	-	-	33.09	-	40	-	-	32.01	-
16	-	-	30.07	-	41	-	-	32.58	-
17	-	-	30.79	-	42	-	-	31.99	-
18	-	-	32.98	-	43	-	-	33.26	-
19	-	-	32.87	-	44	-	-	33.12	-
20	-	-	31.43	-	45	-	-	30.94	-
21	-	-	33.31	-	46	-	-	33.47	-
22	-	-	31.14	-	47	-	-	31.59	-
23	-	-	32.68	-	48	-	-	33.42	-
24	-	-	31.56	-	49	-	-	32.12	-
25	-	-	33.07	-	50	-	-	32.30	-

Bt = batch treated; Bnt = batch not treated; T = seed coat; RC = root+cotyledons.

**Table 2:** Contingency table concerning the actual results for the seed coats.

Seed Coats	Infected	Not Infected	Total
Treated	1	49	50
Not treated	50	0	50
Total	51	49	100

**Table 3:** The most extreme scenario for seed coats.

Seed Coats	Infected	Not Infected	Total
Treated	0	50	50
Not treated	50	0	50
Total	50	50	100

**Table 4:** Contingency table regarding the actual experimented results for the root+cotyledons.

Root+Cotyledons	Infected	Not Infected	Total
Treated	0	50	50
Not treated	2	48	50
Total	2	98	100

**Table 5:** Cotyledons and third true leaf Ct values obtained by the RT-qPCR of untreated tomato seeds (only Ct values of positive samples are reported).

Bnt Samples Group	Positive Sample ID	Ct Values	
		Cotyledons	Third True Leaf
1–20	14	30.12	34.90
	20	28.46	33.84
21–40	-	-	-
41–60	56	33.98	-
61–80	-	-	-
81–100	82	29.98	33.71
	94	31.68	34.83
101–120	-	-	-
121–140	-	-	-
141–160	-	-	-
161–180	162	33.78	34.73
181–200	187	32.56	34.51
	189	34.45	-
201–220	213	34.06	-
221–240	-	-	-
241–260	-	-	-
261–280	-	-	-
281–300	-	-	-
301–320	-	-	-
321–340	-	-	-
341–360	349	27.57	32.56
361–380	-	-	-
381–400	-	-	-
401–420	-	-	-
421–440	422	34.22	-
441–460	442	34.09	-
461–480	466	29.87	34.23
481–500	493	33.63	34.82

Bnt = Batch not treated.

**Table 6:** Percentage of seed germination after different thermal and chemical disinfection treatments on ToBRFV-infected seeds.

Treatment		Seed Germination Percentage (%)	
		7 Days Post-Treatment	14 Days Post-Treatment
Thermal-based treatments	ST-80	100	-
	ST-75	67	100
	ST-70	89	100
	ST-65	100	-
Chemical-based treatments	ST-P	94	100
	ST-H	100	-
	ST-A	0	0
	ST-S	88	100

ST = seed treated; 80 = 80 °C; 75 = 75 °C; 70 = 70 °C; 65 = 65 °C; P = 10% trisodium phosphate; H = 4% hydrogen peroxide; A = 2% hydrochloric acid +1.5% sodium hypochlorite; S = 2.5% sodium hypochlorite solution.

**Table 7:** Proportions of infected seeds with respective lower and upper bounds of the confidence intervals (confidence level 0.95).

Heading	ST-80	ST-75	ST-70	ST-65	ST-P	ST-H	ST-A	ST-S
**Proportion infected**	0.6	0.8	1.00	1.00	0.03	1.00	-	0.00
**Lower bound**	0.50	0.72	1.00	1.00	0.00	1.00	-	0.00
**Upper bound**	0.70	0.88	1.00	1.00	0.06	1.00	-	0.00

## Supplementary Materials

The following are available online at https://www.mdpi.com/2223-7747/9/11/1615/s1, Table S1: Ct values obtained by RT-qPCR after different thermal and chemical disinfection treatments on ToBRFV-infected seeds.
