# Supplementary material for: Tomato Brown Rugose Fruit Virus: Seed Transmission Rate and Efficacy of Different Seed Disinfection Treatments
Source: Plants (Basel). 2020 Nov 20;9(11):1615. doi: 10.3390/plants9111615 (PMC7699967; doi:10.3390/plants9111615)
Supplement: Supplementary file 1 [file plants-09-01615-s001.pdf]

Table S1 – Ct values obtained by Real Time RT-PCR after different thermal and chemical disinfection treatments on ToBRFV-infected seeds.

Sample ID	Ct values							
	ST-80	ST-75	ST-70	ST-65	ST-P	ST-H	ST-A	ST-S
1	-	20.85	24.76	24.12	-	25.93	N.A.	-
2	-	23.54	24.92	27.01	-	27.27	N.A.	-
3	26.55	22.58	25.08	24.49	-	26.24	N.A.	-
4	27.68	25.05	24.77	23.62	27.68	26.34	N.A.	-
5	26.05	24.84	25.56	27.98	-	25.63	N.A.	-
6	26.24	20.57	24.96	24.50	-	25.97	N.A.	-
7	-	-	24.99	26.02	-	26.80	N.A.	-
8	-	20.71	25.44	25.05	-	26.54	N.A.	-
9	-	23.79	25.05	23.54	-	25.95	N.A.	-
10	26.05	25.20	25.57	26.88	-	25.44	N.A.	-
11	27.14	-	25.40	26.66	-	26.37	N.A.	-
12	-	24.26	24.95	23.60	-	25.19	N.A.	-
13	27.03	20.67	26.24	26.94	-	25.83	N.A.	-
14	27.06	21.41	24.99	23.87	-	26.55	N.A.	-
15	-	-	26.17	24.37	-	27.16	N.A.	-
16	26.71	-	24.63	25.35	-	25.02	N.A.	-
17	26.12	-	24.65	26.51	-	24.94	N.A.	-
18	26.56	23.84	24.88	25.04	-	27.07	N.A.	-
19	27.22	23.28	26.19	27.06	-	26.14	N.A.	-
20	27.32	20.97	26.20	24.77	-	25.25	N.A.	-
21	27.22	20.60	24.73	26.67	-	27.16	N.A.	-
22	-	22.10	24.85	28.24	-	26.65	N.A.	-
23	26.72	25.15	24.82	25.88	-	27.09	N.A.	-
24	-	23.23	25.35	25.11	-	26.34	N.A.	-
25	26.12	20.31	26.18	26.79	25.83	25.70	N.A.	-
26	25.85	-	25.61	28.23	-	25.49	N.A.	-
27	27.51	20.07	24.91	26.79	-	25.41	N.A.	-
28	26.42	23.46	24.77	26.98	-	25.96	N.A.	-
29	25.85	23.86	25.31	26.41	-	26.34	N.A.	-
30	25.89	-	25.84	23.45	-	25.22	N.A.	-
31	-	24.96	24.55	26.54	-	26.72	N.A.	-
32	27.14	-	25.19	23.81	-	25.37	N.A.	-
33	-	-	24.59	25.90	-	27.22	N.A.	-
34	-	-	26.22	27.89	-	26.51	N.A.	-
35	-	21.09	26.22	25.90	-	27.30	N.A.	-
36	27.46	21.93	25.40	27.75	-	26.57	N.A.	-
37	26.48	20.10	25.38	23.71	-	27.06	N.A.	-
38	27.27	-	26.45	26.69	-	25.20	N.A.	-
39	27.48	22.83	25.53	28.11	-	25.65	N.A.	-
40	27.11	25.02	26.25	23.56	-	26.80	N.A.	-
41	-	20.90	25.04	24.90	-	25.27	N.A.	-
42	25.91	23.05	25.47	27.92	-	26.70	N.A.	-
43	26.75	20.66	25.97	24.60	-	27.12	N.A.	-
44	26.60	21.88	25.47	25.55	-	25.51	N.A.	-
45	26.82	25.57	24.98	24.54	-	25.35	N.A.	-
46	25.98	23.18	24.87	28.26	-	25.53	N.A.	-
47	26.34	24.89	24.72	26.34	-	26.63	N.A.	-
48	27.64	22.89	25.95	25.26	-	26.88	N.A.	-
49	-	24.63	25.16	25.43	-	26.14	N.A.	-
50	27.17	-	25.51	27.99	26.80	26.35	N.A.	-
51	27.04	20.37	26.26	26.99	-	26.43	N.A.	-
52	-	25.69	24.87	26.63	-	25.70	N.A.	-
53	-	21.84	25.83	27.86	-	27.30	N.A.	-

54	25.83	25.18	26.01	23.94	-	25.92	N.A.	-
55	27.08	-	25.25	25.20	-	25.10	N.A.	-
56	26.53	21.15	25.11	24.54	-	26.47	N.A.	-
57	26.13	24.49	25.00	25.82	-	26.06	N.A.	-
58	-	22.57	24.68	28.38	-	25.52	N.A.	-
59	26.92	20.16	25.38	28.05	-	26.58	N.A.	-
60	-	24.94	25.63	27.17	-	25.52	N.A.	-
61	-	24.31	25.40	27.52	-	26.17	N.A.	-
62	-	-	25.95	26.27	-	26.52	N.A.	-
63	-	25.65	24.88	24.47	-	27.02	N.A.	-
64	27.30	20.64	25.25	23.79	-	25.44	N.A.	-
65	26.41	25.83	26.38	25.35	-	27.07	N.A.	-
66	-	-	24.96	23.43	-	26.58	N.A.	-
67	-	22.35	25.71	26.55	-	25.05	N.A.	-
68	-	20.75	25.56	26.68	-	27.08	N.A.	-
69	25.88	25.65	25.78	23.70	-	25.49	N.A.	-
70	-	21.15	26.23	25.35	-	25.80	N.A.	-
71	-	23.64	26.13	24.80	-	25.04	N.A.	-
72	26.24	21.92	26.40	27.38	-	26.55	N.A.	-
73	27.13	22.66	24.67	25.51	-	25.52	N.A.	-
74	-	24.12	25.52	28.35	-	26.01	N.A.	-
75	26.71	20.12	26.04	25.36	-	25.30	N.A.	-
76	27.54	24.84	25.06	24.32	-	27.08	N.A.	-
77	-	-	25.09	26.26	-	26.80	N.A.	-
78	26.92	-	25.54	25.22	-	25.79	N.A.	-
79	26.61	20.54	26.16	26.64	-	26.20	N.A.	-
80	27.12	21.52	24.82	24.51	-	26.28	N.A.	-
81	26.95	22.19	25.55	26.50	-	24.99	N.A.	-
82	27.06	22.83	25.33	27.36	-	25.25	N.A.	-
83	-	23.42	24.98	27.83	-	26.07	N.A.	-
84	26.19	-	24.59	24.27	-	27.23	N.A.	-
85	-	22.31	24.99	23.59	-	26.61	N.A.	-
86	-	22.86	25.88	27.08	-	26.88	N.A.	-
87	-	23.49	24.65	24.17	-	26.89	N.A.	-
88	-	24.83	26.22	27.10	-	27.11	N.A.	-
89	-	25.73	26.23	26.14	-	25.10	N.A.	-
90	27.63	25.26	25.12	24.01	-	26.67	N.A.	-
91	-	25.42	24.69	27.78	-	26.52	N.A.	-
92	-	25.97	25.49	23.91	-	27.31	N.A.	-
93	-	23.59	26.13	26.71	-	27.28	N.A.	-
94	-	23.85	24.75	25.94	-	26.25	N.A.	-
95	26.84	20.64	24.54	24.59	-	27.21	N.A.	-
96	25.98	26.04	25.38	26.35	-	26.30	N.A.	-
97	-	23.68	25.71	25.84	-	26.31	N.A.	-
98	26.32	22.47	24.65	24.11	-	26.98	N.A.	-
99	27.50	-	25.48	24.49	-	25.39	N.A.	-
100	27.15	-	25.42	25.52	-	26.23	N.A.	-

ST= seed treated; **80**= 80 °C; **75**= 75 °C; **70**= 70 °C; **65**= 65 °C; **P**=10% trisodium phosphate; **H**= 4% hydrogen peroxide; **A**= 2% hydrochloric acid + 1.5% sodium hypochlorite; **S**= 2.5% sodium hypochlorite solution.
